# Early Origins of Hypertension: Should Prevention Start Before Birth Using Natural Antioxidants?

**DOI:** 10.3390/antiox9111034

**Published:** 2020-10-23

**Authors:** Chien-Ning Hsu, You-Lin Tain

**Affiliations:** 1 Department of Pharmacy, Kaohsiung Chang Gung Memorial Hospital, Kaohsiung 833, Taiwan; chien_ning_hsu@hotmail.com; 2 School of Pharmacy, Kaohsiung Medical University, Kaohsiung 807, Taiwan; 3 Department of Pediatrics, Kaohsiung Chang Gung Memorial Hospital and Chang Gung University College of Medicine, Kaohsiung 833, Taiwan; 4 Institute for Translational Research in Biomedicine, Kaohsiung Chang Gung Memorial Hospital and Chang Gung University College of Medicine, Kaohsiung 833, Taiwan

**Keywords:** antioxidant, arginine, developmental origins of health and disease (DOHaD), hypertension, melatonin, *N*-acetylcysteine, nitric oxide, oxidative stress, reactive oxygen species, resveratrol

## Abstract

Hypertension may originate in early life. Reactive oxygen species (ROS) generated due to the exposure of adverse in utero conditions causes developmental programming of hypertension. These excessive ROS can be antagonized by molecules which are antioxidants. Prenatal use of natural antioxidants may reverse programming processes and prevent hypertension of developmental origin. In the current review, firstly we document data on the impact of oxidative stress in hypertension of developmental origin. This will be followed by effective natural antioxidants uses starting before birth to prevent hypertension of developmental origin in animal models. It will also discuss evidence for the common mechanisms underlying developmental hypertension and beneficial effects of natural antioxidant interventions used as reprogramming strategies. A better understanding of the reprogramming effects of natural antioxidants and their interactions with common mechanisms underlying developmental hypertension is essential. Therefore, pregnant mothers and their children can benefit from natural antioxidant supplementation during pregnancy in order to reduce their risk for hypertension later in life.

## 1. Introduction

One in three adults across the globe have high blood pressure (BP), known as hypertension [1]. Despite recent advances in the treatment of hypertension, raised BP remains one of the leading causes of morbidity worldwide [2]. Growing evidence indicates that the origins of hypertension can begin in early life [3,4,5]. This theory, now called “developmental origins of health and disease (DOHaD)”, is based on observing the developing fetus, if in utero exposed to an adverse environment, increases risk for developing adult diseases later in life [6]. In order to reduce the global burden of hypertension, we need to ascertain the mechanisms underlying the early origins of hypertension and provide strategies for early detection and intervention.

The imbalance between reactive oxygen species (ROS) production and antioxidants defense system causes oxidative stress and plays a pathophysiological role in fetal programming [7]. Cumulative evidence has shown that oxidative stress, experienced early in life, increases a later risk of hypertension [8,9,10]. Conversely, the use of antioxidant supplements during the period of developmental plasticity [10,11,12] may be beneficial in reversing the programming processes to prevent adult diseases, also known as reprogramming [4,13]. Therefore, this review aims to address the main scientific findings on the interplay among oxidative stress, natural antioxidants, and developmental programming in hypertension.

Particular interest in this review is that we highlighted the use of natural antioxidants as reprogramming strategies, in order to prevent developmental hypertension via reversing programming processes. The PubMed/MEDLINE database was used to identify related peer-reviewed journal articles published in English. Additional studies were selected based on references from eligible articles. We used different combinations of keywords as follows: “antioxidants”, “hypertension”, “blood pressure”, “developmental programming”, “DOHaD”, “free radicals”, “offspring”, “melatonin”, “mother”, “nitric oxide”, “oxidative stress”, “pregnancy”, “progeny”, “reprogramming”, “reactive oxygen species”, “reactive nitrogen species”, “resveratrol”, and “vitamin”. The last search was conducted on 15 September 2020.

## 2. Oxidative Stress and Developmental Programming of Hypertension

### 2.1. Oxidative Stress in Pregnancy

Fetal oxygen requirements vary throughout pregnancy [7]. During the first trimester, fetal oxygen levels are low. Low physiological oxygen tension is required for early differentiation and organogenesis. The increased oxygen levels that occur as a result of the establishment of the fetal–placental circulation allows rapid gain of fetal weight during the second and third trimesters [14]. Many maternal conditions cause increased oxidative stress, such as diabetes, obesity, preeclampsia, and smoking [7,8]. Accordingly, ROS plays dual behavior in pregnancy, as produced at high level negatively affects fetal development while moderate amount is essential to allow for the normal embryonic and fetal growth [7].

**Figure 1:**
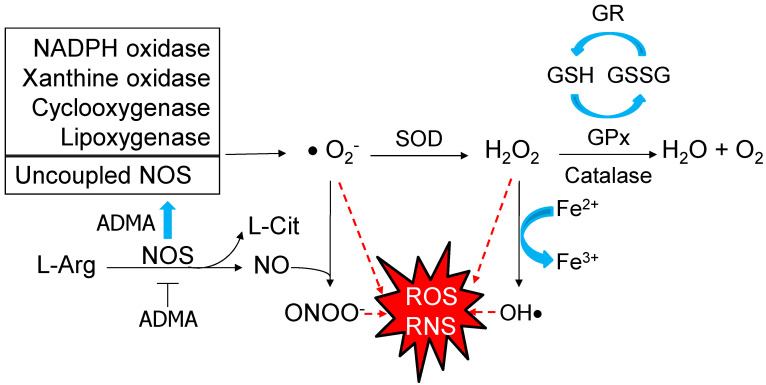
Schematic representation of the pathways producing reactive oxygen species (ROS)/reactive nitrogen species (RNS) and key defensive antioxidant enzymatic systems. Several enzymes produce superoxide radical (O_2_^−^), such as NADPH oxidase, xanthine oxidase, cyclo-oxygenase, lipoxygenase, and uncoupled nitric oxide synthase (NOS). NOS catalyzes L-arginine (L-Arg) to produce nitric oxide (NO) and L-citrulline (L-Cit). While being inhibited by asymmetric dimethylarginine (ADMA), uncoupled NOS generates superoxide instead of NO. High level of NO can interact with superoxide to form peroxynitrite (ONOO^−^). Red dashed arrow lines indicate O_2_^−^, hydrogen peroxide (H_2_O_2_), hydroxyl anion (OH^−^), and peroxynitrite (ONOO^−^) are key elements of ROS/RNS. Conversely, excessive ROS/RNS can be counterbalanced by various antioxidant enzymes, such as superoxide dismutase (SOD), catalase, glutathione peroxidase (GPx), and glutathione reductase (GR). GPx converts reduced glutathione (GSH) into oxidized glutathione (GSSG). The generated GSSG is reduced to GSH with consumption of NADPH by GR.

The formation of superoxide anion (O_2_^−^) leads to a cascade of other ROS, like hydrogen peroxide (H_2_O_2_) and hydroxyl anion (OH^−^). Several enzymes such as nicotinamide adenine dinucleotide phosphate (NADPH) oxidases, xanthine oxidase, cyclo-oxygenases, and lipoxygenases can produce superoxide radical [15]. Superoxide radical can also be generated by uncoupled nitric oxide synthases (NOS) in certain conditions, like inhibition by asymmetric dimethylarginine (ADMA) [16]. Generally, NOS produces nitric oxide (NO), a free radical, as well as a vasodilator. The NO has dual role in pregnancy which depends on its concentration. High levels of NO can interact with superoxide to form peroxynitrite (ONOO^−^), a most injurious reactive nitrogen species (RNS) with pronounced deleterious effects. Conversely, maintained physiological level of NO is required during healthy normal pregnancy [17]. The excessive ROS can be neutralized by enhancing defense antioxidant system, including superoxide dismutase (SOD), catalase, glutathione peroxidase (GPx), and glutathione reductase (GR) [18]. In compromised pregnancy, oxidative damage occurs because of the failure of defensive antioxidant mechanisms to respond to the excessive ROS, leading to DNA damage, lipid peroxidation, protein modification, and mitochondrial dysfunction [18]. These processes are involved in the pathogenesis of developmental programming of hypertension. The main pathways producing ROS/RNS and key defensive antioxidant enzymatic systems are illustrated in Figure 1.

### 2.2. Developmental Programming of Hypertension

Important support for the developmental programming of hypertension came from human and experimental studies. Several risks associated with high BP of offspring have been identified in mother-child cohorts, including maternal undernutrition [19], maternal obesity [20], short term breastfeeding [21], maternal smoking [22], gestational hypertension [23], low vitamin D consumption [24], and excessive postnatal weight gain [25]. A meta-analysis of 1342 preterm or very low birth weight (VLBW) and 1738 full term individuals reported that those born preterm or VLBW have modestly higher BP later in life [26].

Although, human observational studies cannot establish direct cause-and-effect relationships between adverse maternal environmental factors and offspring hypertension, where emerging evidence from animal studies have confirmed the types of prenatal insults that drive disease programming and identify potential mechanisms. As previously reviewed by us and others [5,27,28,29], maternal malnutrition, maternal illness like diabetes, perinatal hypoxia, environmental chemicals, toxins, and the use of medication in pregnancy have all been reported to affect developmental programming and increase the risk for developing hypertension in adulthood.

**Figure 2:**
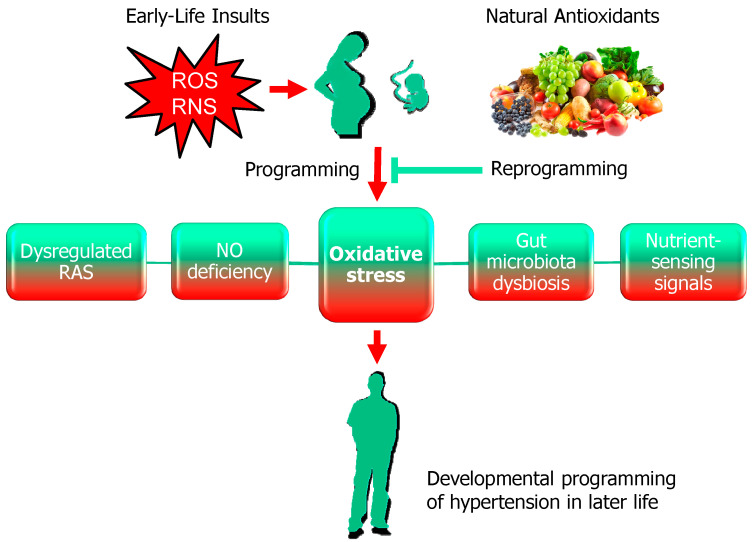
Schematic illustration of the impact of oxidative stress and natural antioxidants on hypertension of developmental origin. Red arrow indicates early-life insults in pregnancy causes increased reactive oxygen species (ROS)/reactive nitrogen species (RNS) and fetal programming, consequently resulting in hypertension in adult offspring. Oxidative stress acts as a central hub through which mechanisms contributing to programming hypertension are interconnected. These mechanisms include dysregulated the renin-angiotensin system (RAS), nitric oxide (NO) deficiency, gut microbiota dysbiosis, and impaired nutrient-sensing signals. Conversely, natural antioxidants can serve as reprogramming strategies to reverse the programmed processes and prevent the developmental programming of hypertension, which is indicated by green T-bar lines.

Current evidence suggests that there may be common mechanisms underlying hypertension of developmental origin. Animal models have provided significant insight into the molecular mechanisms, such as oxidative stress, dysregulation of the renin-angiotensin system (RAS), impaired nutrient-sensing signals, NO deficiency, and gut microbiota dysbiosis [5,27,28,29]. Among them, oxidative stress plays a crucial role and is closely linked to other important mechanisms involved in programmed hypertension (Figure 2).

### 2.3. The Impact of Oxidative Stress in Hypertension of Developmental Origin

Several lines of evidence support the role of oxidative stress in the developmental programming of hypertension. First, cumulative evidence indicates that hypertension, programmed by various early-life insults, are associated with oxidative stress, as reviewed elsewhere [5,10]. These adverse perinatal environmental conditions include maternal caloric restriction [30], maternal diabetes [31], maternal nicotine exposure [32], ethanol consumption [33], preeclampsia [34], high-fat diet [35], high-fructose consumption [36], high-salt diet [37], methyl-donor diet [38], iron deficient diet [39], zinc deficient diet [40], magnesium deficient diet [41], prenatal glucocorticoid exposure [42], prenatal hypoxia [43], and exposure to environmental chemicals [44,45]. Second, there are reports that ADMA levels, a NOS inhibitor and ROS inducer, are associated with the elevation of BP in various developmental animal models [30,31,34]. Conversely, early interventions lowering ADMA levels and restoring NO-ROS balance can protect adult offspring against hypertension [10]. Another line of evidence comes from studies of antioxidant system and oxidative stress damage markers. Our previous study reported that adult offspring born to dams that have received low protein diet developed hypertension, which was associated with decreased antioxidant glutathione level and increased 8-isoprostaglandin F2α level (a biomarker of lipid peroxidation) [46]. Maternal high-fat diet caused raised BP in adult offspring related to increased malondialdehyde levels together with decreased antioxidant enzyme activities of SOD, GPx, and catalase [47]. Additionally, increased oxidative DNA damage marker 8-hydroxydeoxyguanosine (8-OHdG) expression has been reported in several models of programmed hypertension [35,38,48]. Together these observations indicate that oxidative stress is an important pathogenetic link for hypertension of developmental origin.

## 3. Natural Antioxidants

### 3.1. Natural Antioxidants and Hypertension

Antioxidants can be categorized as enzymatic antioxidants and nonenzymatic antioxidants. The human body protects itself from the harmful effects of ROS by using enzymatic antioxidants to modulate the free radical reactions. There are two non-enzymatic antioxidants, the natural antioxidants and the synthetic antioxidants [49]. Given that the scope of this article is limited to the natural antioxidants, we will not discuss the synthetic antioxidants.

Natural antioxidants are mainly coming from plants, such as vegetables, fruits, nuts, and seeds. Antioxidants obtained from vegetables and fruits are mainly phenolic compounds, and the most important are the polyphenols, vitamins, minerals, and flavonoids [50]. Therefore, dietary sources are very important since they can be easily used for dietary interventions.

Randomized clinical trials employing nonpharmacological dietary interventions emphasizing dietary antioxidant nutrients have shown notable BP-lowering results in hypertensive and normotensive subjects [51,52,53]. The dietary components in these studies are high in compounds known as natural antioxidants. As reviewed elsewhere [54], these commonly used antioxidants include Vitamins A, C and E, L-arginine, flavonoids, coenzyme Q10, β-carotene, and α-lipoic acid. However, so far specific natural antioxidants are not yet recommended for antihypertensive therapy due to lack of target specificity, lack of understanding of action mechanisms, and potential interindividual variability in therapeutic efficacy [54]. Additionally, melatonin [55], resveratrol [56], and *N*-acetylcysteine [57] all have shown antihypertensive effects through antioxidant mechanisms counterbalancing excessive ROS. Some of the natural antioxidants that have been isolated from various natural sources are shown in the Table 1.

### 3.2. Natural Antioxidants as Reprogramming Interventions

Despite dietary and nutritional supplements during pregnancy and lactation have been recommended for improving maternal and newborn health and survival [68,69]. Little is known on whether supplementing with specific natural antioxidants, starting before birth, can be beneficial on hypertension programmed by adverse maternal conditions in humans. Here, we summarize the knowledge available today regarding natural antioxidants used as reprogramming strategies for developmental hypertension in various animal models [30,31,38,44,70,71,72,73,74,75,76,77,78,79,80,81,82,83,84,85,86,87,88,89,90,91,92,93,94,95,96,97,98], all of which are listed in Table 2. We restricted this review to natural antioxidants applied only during pregnancy and/or lactation which are critical periods for reprogramming strategies to prevent the development of hypertension. So far, many natural antioxidants have shown benefits on prevention of developmental hypertension, such as amino acids, vitamins and minerals, melatonin, resveratrol, and *N*-acetylcysteine. In this review, rats are the commonly used small animal models. Rats develop rapidly during infancy and reach sexual maturity at approximately 5–6 weeks of age. In adulthood, one human year almost equals two rat weeks [99]. Accordingly, Table 2 lists the outcomes determined in rats ranging from 4 to 50 weeks of rat age, which allows calculations to extract data for the specific age group that can be translated to humans. However, very limited information is available regarding large animals to study the role of natural antioxidants on hypertension of developmental origin.

### 3.3. Amino Acids

Several amino acids have antioxidant properties [100]. Some of them have been reported to regulate BP [101]. L-glycine supplementation during pregnancy and lactation was shown to protect against maternal low-protein intake-induced programmed hypertension in offspring [70], which agrees well with a previous study demonstrating that glycine administration produced depressor responses on BP [100].

A decreased NO bioavailability is one of the pathogenetic mechanisms underlying hypertension of developmental origin [102]. Both L-arginine (the substrate for NO synthase) and L-citrulline, a precursor of L-arginine, can be supplemented to increase NO production [103]. As shown in Table 2, perinatal L-citrulline supplementation had a beneficial effect on offspring BP in a variety of animal models, including maternal streptozotocin (STZ)-induced diabetes [31], Maternal N^G^-nitro–L-arginine methyl ester (L-NAME) exposure [71], and prenatal dexamethasone exposure [72]. In spontaneously hypertensive rats (SHR), perinatal supplementation with L-citrulline can prevent the transition from prehypertension to hypertension via restoration of NO bioavailability [73]. However, whether L-arginine supplementation in pregnancy alone is associated with these beneficial effects has not been clarified.

Additionally, L-taurine has been used alone or combined with other antioxidants to prevent hypertension programmed by a variety of early-life insults [74,75,78,79,80]. L-taurine is a common sulfur-containing amino acid [104]. Several beneficial effects of L-taurine on hypertension have been reported, including regulation of NO and hydrogen sulfide (H_2_S), regulation of the renin–angiotensin system (RAS), and reduction of oxidative stress [105,106]. Perinatal L-taurine use showed protection in hypertension programmed by maternal high-sugar consumption or maternal STZ-induced diabetes [74,75]. A combination of L-taurine, L-arginine, and Vitamins C and E therapy in the perinatal period caused a reduction of BP in SHRs and in Fawn hooded hypertensive rats (FHH), two genetic models of hypertension [78,79,80]. Furthermore, other amino acids, like L-tryptophan [76] and branched-chain amino acids (BCAAs) [77], have been used as reprogramming interventions, by which hypertension could be prevented in adult offspring. Despite amino acids with antioxidant properties have been increasingly investigated for their reprogramming benefits on hypertension of developmental origin, there is still an unmet need in better understanding the accurate dietary recommendations for these amino acids for pregnant women in normal and compromised pregnancy.

### 3.4. Vitamins

Vitamin C, E, folic acid and selenium, which showed a beneficial effect on BP in established hypertension [107], were also considered as potential protective compounds against hypertension of developmental origin. Vitamins C and E are the most frequently used antioxidant vitamins. Vitamin C is a six-carbon lactone with the ability of ROS quenching. Vitamin E (α-Tocopherol) is a fat-soluble carotenoid that inhibits NADPH oxidase, lipoxygenase, and cyclooxygenase [108]. Gestational use of Vitamin C or E alone protected the elevation of BP in adult male offspring exposed to prenatal lipopolysaccharide (LPS) [82,84]. Also, the combined supplementation of vitamins C, E, folic acid, and selenium can prevent hypertension programmed by maternal caloric restriction [81]. Moreover, maternal supplementation with folic acid can prevent offspring against hypertension in a maternal low protein diet model [83].

However, The Cochrane Collaboration compiled 56 clinical trials that included almost a quarter million participants to conclude that β-carotene, vitamin E, and high doses of vitamin A seem to increase mortality [109]. Although excessive dietary vitamin A intake has been associated with birth defects in humans [110], whether excessive vitamin supplementation affects hypertension reprogramming remains largely unknown.

### 3.5. Melatonin

Melatonin, an endogenous indoleamine derived from tryptophan, has pleiotropic biofunctions, such as antioxidant, anti-inflammation, regulation of circadian rhythm, epigenetic regulation, and fetal development [111,112,113,114,115]. As reviewed elsewhere [12], melatonin has emerged as a common reprogramming strategy to prevent many adult diseases in different models of developmental programming. Several mechanisms, including reduction of oxidative stress, restoration of NO, epigenetic regulation, and rebalancing the RAS have been associated with the reprogramming effects of melatonin [12].

As an antioxidant, not only melatonin but also its metabolites have abilities to scavenge ROS and RNS [115]. Table 2 shows perinatal melatonin therapy prevents hypertension programmed by diverse early-life insults, such as maternal caloric restriction [30], maternal methyl-donor diet [38], maternal constant light exposure [85], maternal L-NAME exposure [86], high-fructose diet [87], high-fructose diet plus post-weaning high-salt diet [88], prenatal dexamethasone exposure [89], and prenatal dexamethasone exposure plus post-weaning high-fat diet [90]. Additionally, maternal melatonin therapy can prevent the transition from prehypertension to hypertension in SHRs [91]. The protective effects of maternal melatonin therapy against hypertension are associated with increased NO bioavailability [30,38,87,88], reduced ADMA levels [30,88], decreased 8-OHdG expression [38], and decreased 8-isoprostane level [86]. Together, these findings emphasize that melatonin works as an antioxidant in different ways to benefit hypertension reprogramming.

So far, serious adverse events are scarce in humans receiving melatonin treatment ranged from 0.3 mg to 1600 mg daily [112,116,117]. Although, melatonin has a generally favorable safety profile, no clinical trials of melatonin in pregnant women have been identified to assess its use and safety. A previous study demonstrated that pregnant sheep received a high dose of melatonin and its levels were raised up to 200 times normal values, leading to no adverse effect on fetal health [118]. It is noteworthy that maternal melatonin treatment can cause long-term transcriptomic changes and regulate numerous biological pathways [113]. Whether these programmed processes and pathways might be interconnected with its antioxidant mechanism to prevent programmed hypertension remains to be elucidated.

### 3.6. Resveratrol

Polyphenols include anthocyanins, flavonoids, and stilbenes [50]. Resveratrol is a natural polyphenol from the stilbene family that occurs as a phytoalexin [119]. Resveratrol constitutes functional foods with many health benefits [120]. Resveratrol exerts pleiotropic functions including anti-inflammatory and antioxidant properties, improvement of endothelial function, anti-atherosclerotic and anti-obesogenic effect, anticarcinogenic activity, and restoring bioavailable NO production [121]. The antioxidant effects of resveratrol include ROS/RNA scavenging ability, enhancement of various antioxidant defensive enzymes, induction of glutathione level, increases of NO bioavailability, and activation of nuclear factor-erythroid 2-related factor 2 (Nrf2) [122,123].

Currently, many human studies have reported that resveratrol is a well-tolerated and safe supplement [121,122,124]. However, others have shown toxic effects of resveratrol in vitro and in vivo [125]. Resveratrol appears to have a hormetic effect where resveratrol like an antioxidant at low doses are associated with beneficial effects, while a pro-oxidant at high doses usually have a toxic effect [126]. However, limited data are available regarding the effects of resveratrol supplementation during pregnancy on offspring’s health [123].

Table 2 indicates reprogramming effects of maternal resveratrol therapy on offspring’s hypertension in rats ranging from 12 to 20 weeks of age [44,45,82,83,84]. However, its long-term effect on offspring outcome remains largely unknown. In a maternal combined bisphenol A exposure and high-fat diet model, the protective effects of maternal resveratrol treatment against offspring hypertension are associated with increased NO bioavailability and decreased renal 8-OHdG expression [38]. Similarly, perinatal resveratrol use can restore the balance between ROS and NO to protect against hypertension in adult offspring born to dams exposed to combined TCDD and dexamethasone administration [45], high-fructose diet [92], and L-NAME plus postnatal high-fat diet [93]. Additionally, the high BP in adult SHRs can also be prevented by resveratrol supplementation in pregnancy and lactation [94].

### 3.7. N-Acetylcysteine

*N*-acetylcysteine (NAC), a plant antioxidant naturally found in onion, is a precursor to glutathione [127]. NAC is also a stable L-cysteine analogue and can be a precursor for H_2_S synthesis. H_2_S is a gaseous signaling molecule with antihypertensive properties [128]. Accordingly, NAC has been reported to prevent hypertension in human trials and experimental studies [129,130]. As shown in Table 2, the beneficial effects of maternal NAC therapy on hypertension reprogramming have been reported in a variety of animal models, including maternal nicotine exposure [32], suramin-induced pre-eclampsia [34], L-NAME exposure [84], and prenatal dexamethasone and postnatal high-fat diet [95]. In a prenatal dexamethasone and postnatal high-fat diet model, the beneficial effects of NAC against offspring hypertension were linked to an increase in plasma glutathione level and H_2_S-generating enzymes, and reduction of oxidative stress [95]. In another study, perinatal use of NAC also protected offspring against hypertension programmed by maternal L-NAME exposure via increases of H2S-generating enzymes and activity in offspring kidneys [84]. Moreover, maternal NAC therapy prevented programmed hypertension in adult offspring born to suramin-treated females was associated with increased glutathione levels, restoration of NO and H_2_S pathways [34].

### 3.8. Others

Conjugated linoleic acid (CLA), a functional lipid with hypotensive and antioxidant activity, have attracted increasing interest recently for its health benefits [131]. One report demonstrated that perinatal CLA supplementation attenuated hypertension programmed by maternal high-fat consumption in adult male offspring [96]. Fish oil is a dietary source of omega-3 polyunsaturated fatty acids, which act like an antioxidant with health benefits [132]. Although current evidence suggests that mega-3 polyunsaturated fatty acid could prevent the rise in BP in hypertensive subjects [133], only one study reported that maternal fish oil supplementation was capable of protecting adult offspring against hypertension programmed by maternal low protein diet [97]. Regardless of BP-lowering effects of supplementing natural antioxidants in lactation, such as melinjo (Gnetum gnemon) seed extract [134], grape skin extract [47], and 15-deoxy-Δ12,14 -prostaglandin J2 (15d-PGJ2) [48], have been reported, all of them have not yet been examined in pregnancy.

It is noteworthy that keeping a physiological oxidative-antioxidative balance is advised to prevent hypertension of developmental origin. Excess antioxidants may shift oxidative stress to an opposite state of “antioxidant stress” [135]. Therefore, a critical balance of antioxidants intake needs to be assessed for the clinical situation to avoid their adverse effects. The intake of natural antioxidant supplements would only make sense in a case of deficits, trying to restore their levels, but not as a usual intake [136].

## 4. Protective Role of Natural Antioxidants on Common Mechanisms Involved in Programmed Hypertension

The primordial studies in animal models with controlled interventions provided important results revealing potential protective mechanisms of natural antioxidants against developmental hypertension. These beneficial mechanisms of natural antioxidants on programmed hypertension include restoration of ADMA-NO pathway, rebalancing of the RAS, activation of nutrient-sensing signals, and reshaping gut microbiota (Figure 2).

### 4.1. Restoration of ADMA-NO Pathway

ADMA-induced NO–ROS imbalance is involved in the development of hypertension, while restoration of the ADMA-NO balance has been considered a potential reprogramming strategy for hypertension of developmental origin [101,137]. Due to multiple metabolic fates, L-arginine is not considered as a good NO precursor [101]. Unlike L-arginine, L-citrulline can bypass hepatic metabolism and it is not a substrate of arginase. As we mentioned earlier, maternal supplementing with L-citrulline can protect adult offspring against the developmental programming of hypertension via restoration of NO bioavailability in several animal models [31,71,72,73].

Additionally, several natural antioxidants have been reported to lower ADMA levels and restore NO-ROS balance in human and experimental studies [101,137,138]. These antioxidants include vitamin E, salvianolic acid A, melatonin, resveratrol, *N*-acetylcysteine, oxymatrine, and epigallocatechin-3-gallate. However, only few ADMA-lowering antioxidants have been examined for prevention of hypertension in the developmental programming models, like resveratrol [92,93], melatonin [85,86,87], and NAC [34,84]. Similar to programming hypertension models, melatonin [139] and NAC [140] have been revealed to prevent the development of hypertension in SHRs by decreasing plasma ADMA levels. Currently, however, a specific ADMA-lowering antioxidant is not available in clinical practice. A therapeutic approach to restore bioavailable NO production by lowering ADMA, thereby preventing the development of hypertension still awaits further evaluation.

### 4.2. Rebalancing of the Renin-Angiotensin System

RAS is a coordinated hormonal cascade in the regulation of BP. The classical RAS comprises the angiotensin converting enzyme (ACE)-Ang II-angiotensin type 1 receptor (AT1R) axis that promotes the elevation of BP. Pharmacological therapies based on the blockade of classical RAS are used extensively for the treatment of hypertension [141]. Emerging evidence supports the theory that dysregulated RAS is a common mechanism underlying programmed hypertension [10,13,142]. Conversely, early blockade of the classical RAS can reprogram inappropriate activation of the RAS, thereby prevention of developmental hypertension [143,144].

Several lines of evidence support that rebalancing the RAS by natural antioxidants has impact on developmental hypertension. First, one report demonstrated that resveratrol therapy protected adult offspring against hypertension programmed by maternal plus post-weaning high-fat diet. Its protective effects were associated with increased plasma Ang (1–7) level and decreased plasma Ang II level [145]. Secondly, maternal L-NAME exposure-induced increases of renal renin and ACE expression was prevented by maternal NAC therapy [84]. Thirdly, there are studies showing that the beneficial effects of melatonin are related to increased ACE2 level. ACE2 belongs to the non-classical RAS pathway, which appears to antagonize the effects of the classical RAS [141]. A previous study from our laboratory examined the maternal caloric restriction-induced hypertension model and found maternal melatonin therapy protected offspring against hypertension, which is related to increased renal ACE2 expression [30]. Likewise, melatonin therapy was reported to prevent the development of offspring’s hypertension was associated with increased ACE2 level in a maternal light exposure model [85]. Last, adult male offspring exposed to prenatal dexamethasone exposure and postnatal high-fat diet developed hypertension; this was associated with increased oxidative stress and activation of the classical RAS [146]. However, Nrf2 activation therapy in pregnancy not only prevented the rising BP but also reduced oxidative stress and downregulated the classical RAS concurrently [146].

### 4.3. Activation of Nutrient-Sensing Signals

Nutrient-sensing signaling pathways are commonly deregulated in fetal development and programmed hypertension, as reviewed elsewhere [10]. Cyclic adenosine monophosphate (AMP)-activated protein kinase (AMPK), silent information regulator T1 (SIRT1), peroxisome proliferator-activated receptors (PPARs), and PPARγ coactivator-1α (PGC-1α), are well-known nutrient-sensing signals [147].

Nutrient-sensing signals regulate PPARs and their target genes, consequently leading to developmental hypertension [148]. There are several PPAR target genes, such as *Sod2*, *Nos2*, *Nos3*, and *Nrf2*, involved in oxidative stress [149]. A variety of early-life insults can downregulate nutrient-sensing signals, to induce hypertension of developmental origin. These adverse in utero environmental conditions include maternal methyl donor diet [38], maternal high-fructose plus post-weaning high-salt diets [88], high-fructose diet [92], high-fat diet [150], and maternal L-NAME exposure and post-weaning high-fat diet [93]. Conversely, interventions activating the AMPK/SIRT1/PGC-1α pathway has shown beneficial on hypertension reprogramming [151]. Because nutrient-sensing signals are interconnected with redox regulation, AMPK plays an important role in regulating antioxidant defense during oxidative stress [151]. The reprogramming effects against hypertension by gestational supplementation of natural antioxidants, such as melatonin [88] and resveratrol [93] were related to upregulate several nutrient-sensing signals.

### 4.4. Reshaping Gut Microbiota

Gut microbiota regulates the cellular redox state within the host organism. In the gut, microbes-mediated ROS production in low levels maintains gut homeostasis, whereas high levels of ROS lead to oxidative stress damage [152]. On the other hand, gut microbe-mediated therapies have been applied as a therapeutic approach for several oxidative stress-associated diseases [153].

Early-life gut microbiota dysbiosis adversely affects fetal programming and may have a long-range negative influence on adult health outcomes [154]. The gut microbiota produces a variety of metabolites, including antioxidant vitamins [155]. Gut microbiota dysbiosis has been linked to hypertension related to several mechanisms, including alterations of microbial metabolites, activation of the RAS, inhibition of NO, increased sympathetic activity, and mediation of the H_2_S signaling pathway [156].

Emerging evidence supports the notion that gut microbiota dysbiosis in early-life is correlated with hypertension of developmental origin [76,86,96,150,157,158]. Dietary fiber intake for modulating the microbiota has become one dietary strategy. Our recent reports showed that prebiotic inulin (i.e., a special form of dietary fiber) supplementation during pregnancy and lactation can protect offspring against hypertension programmed by maternal high-fructose or high-fat consumption [157,158]. Another study from our laboratory examined the high-fat diet-induced hypertension model and found that modulation of gut microbiota by resveratrol can protect adult offspring against programmed hypertension and oxidative stress concurrently [44]. Although, recent studies have demonstrated that probiotics and prebiotics have antioxidants property [152,153,159], their roles in oxidative stress-related hypertension of developmental origin, especially their use in pregnancy, require further investigation.

Together, natural antioxidant interventions in pregnancy may reprogram common mechanisms to prevent offspring against hypertension of developmental origin. However, these effects await further efforts to bridge gaps between basic animal research and clinical translation.

## 5. Conclusions

This review recapitulates that the use of effective natural antioxidants starting before birth protects adult offspring against hypertension in various animal models of developmental programming. However, natural antioxidants can also be disadvantageous. Yet, at the same time, we are aware that a long road still lies ahead in determining the right dose of natural antioxidant for the right person, at the right time, for clinical applications. Further research will help to better delineate the mechanisms underlying developmental hypertension by which these processes occur, and whether specific natural antioxidant therapies are implemented in humans to obviate the global burden of hypertension.

## Figures and Tables

**Table 1:** Different sources of natural antioxidants.

Antioxidants	Natural Sources	References
Vitamin A	Meat, fish, fruits, and vegetables	[58]
Vitamin C	Most fruits and some vegetables, particularly citrus fruits, and tomatoes	[59,60]
Vitamin E	Vegetables oils, nuts, broccoli, and fish	[60,61]
L-arginine	Meat, dairy products, eggs, nuts, and seeds	[62]
Flavonoids	Potatoes, tomatoes, lettuce, onions, wheat, dark chocolate, concord grapes, and black tea	[60,61]
α-lipoic acid	Yeast, organ meats, spinach, broccoli, and potatoes	[63]
β-carotene	Kale, red paprika, spinach, parsley, tomatoes, and carrots	[64]
Coenzyme Q10	Wheat bran, fish, and organ meats	[60,64]
Melatonin	Eggs, meat, fish, milk, nuts, seeds, cereals, peppers, tomatoes, and mushrooms	[65]
Resveratrol	Grapes, peanuts, cocoa, soy, and berries	[66]
*N*-acetylcysteine	Chicken, turkey, garlic, yogurt, and eggs	[67]

**Table 2:** Animal models dealing with natural antioxidants for hypertension reprogramming.

Natural Antioxidants	Animal Models	Intervention Period	Species/ Gender	Age at BP Determination (week)	Beneficial Effects	Ref.
Amino acids						
3% L-glycine in chow	Maternal low protein diet	Pregnancy and lactation	Wistar/M	4	Prevented hypertension	[70]
0.25% L-citrulline in drinking water	Maternal STZ-induced diabetes	Pregnancy and lactation	SD/M	12	Prevented hypertension	[31]
0.25% L-citrulline in drinking water	Maternal L-NAME exposure	Pregnancy and lactation	SD/M	12	Prevented hypertension	[71]
0.25% L-citrulline in drinking water	Prenatal dexamethasone exposure	Pregnancy and lactation	SD/M	12	Prevented hypertension	[72]
0.25% L-citrulline in drinking water	Genetic hypertension	2 weeks before until 6 weeks after birth	SHR/M & F	50	Prevented hypertension	[73]
3% L-taurine in drinking water	Maternal highsugar diet	Pregnancy and lactation	SD/F	8	Prevented hypertension	[74]
3% L-taurine in drinking water	Maternal STZ-induced diabetes	Pregnancy and lactation	Wistar/M & F	16	Prevented hypertension	[75]
L-tryptophan 200 mg/kg BW/day via oral gavage	Maternal adenosine-induced CKD	Pregnancy	SD/M	12	Prevented hypertension	[76]
BCAA-supplemented diets	Maternal caloric Restriction	Pregnancy	SD/M	16	Prevented hypertension	[77]
Amino acids plus vitamins						
L-arginine, L-taurine, Vitamins C and E	Genetic hypertension	2 weeks before until 8 weeks after birth	SHR/M& F	9	Prevented hypertension	[78]
L-arginine, L-taurine, Vitamins C and E	Genetic hypertension	2 weeks before until 4 weeks after birth	FHH/M & F	36	Prevented hypertension	[79]
L-arginine, L-taurine, Vitamins C and E	Genetic hypertension	2 weeks before until 8 weeks after birth	SHR/M & F	50	Prevented hypertension	[80]
Vitamins						
Vitamin C, E, folic acid and selenium	Maternal caloric Restriction	Pregnancy	Wistar/ M & F	16	Prevented hypertension	[81]
Vitamin C 350 mg/kg/day i.p. daily	Prenatal LPS Exposure	Gestational day 8 to 14	SD/M	12	Prevented hypertension	[82]
5 mg/kg folate in chow	Maternal low protein diet	Pregnancy	Wistar/M	15	Prevented hypertension	[83]
α-tocopherol 350 mg/kg/day via gavage	Prenatal LPS Exposure	Gestational day 13 to 20	Wistar/M	28	Prevented hypertension	[84]
Melatonin						
0.01% melatonin in drinking water	Maternal caloric restriction	Pregnancy and lactation	SD/M	12	Prevented hypertension	[30]
0.01% melatonin in drinking water	Maternal methyl-donor diet	Pregnancy and lactation	SD/M	12	Attenuated hypertension	[38]
0.01% melatonin in drinking water	Maternal constant light exposure	Pregnancy and lactation	SD/M	12	Prevented hypertension	[85]
0.01% melatonin in drinking water	Maternal L-NAME exposure	Pregnancy and lactation	SD/M	12	Prevented hypertension	[86]
0.01% melatonin in drinking water	Maternal high-fructose diet	Pregnancy and lactation	SD/M	12	Prevented hypertension	[87]
0.01% melatonin in drinking water	Maternal high-fructose diet plus post-weaning high-salt diet	Pregnancy and lactation	SD/M	12	Attenuated hypertension	[88]
0.01% melatonin in drinking water	Prenatal dexamethasone exposure	Pregnancy and lactation	SD/M	16	Prevented hypertension	[89]
0.01% melatonin in drinking water	Prenatal dexamethasone exposure plus post-weaning high-fat diet	Pregnancy and lactation	SD/M	16	Prevented hypertension	[90]
Melatonin 10 mg/kg BW/day in drinking water	Genetic hypertension model	Pregnancy	SHR/M	16	Prevented hypertension	[91]
Resveratrol						
50 mg/L resveratrol in drinking water	Maternal plus post-weaning high-fructose diet	Pregnancy and lactation	SD rat/M	12	Prevented hypertension	[92]
50 mg/L resveratrol in drinking water	Maternal bisphenol A exposure and high-fat diet	Pregnancy and lactation	SD rat/M	16	Prevented hypertension	[44]
0.05% resveratrol in drinking water	Maternal TCDD and dexamethasone exposures	Pregnancy and lactation	SD rat/M	16	Prevented hypertension	[45]
50 mg/L resveratrol in drinking water	Maternal L-NAME plus postnatal high-fat diet	Pregnancy and lactation	SD rat/M	16	Attenuated hypertension	[93]
4 g/kg diet resveratrol	Genetic hypertension model	Pregnancy and lactation	SHR/M & F	20	Prevented hypertension	[94]
*N*-acetylcysteine (NAC)						
1% NAC in drinking water	Suramin-induced preeclampsia	Pregnancy and lactation	SD/M	12	Prevented hypertension	[34]
1% NAC in drinking water	Maternal L-NAME exposure	Pregnancy and lactation	SD/M	12	Prevented hypertension	[84]
1% NAC in drinking water	Prenatal dexamethasone and postnatal high-fat diet	Pregnancy and lactation	SD/M	12	Prevented hypertension	[95]
1% NAC in drinking water	Genetic hypertension model	Pregnancy and lactation	SHR/M	12	Prevented hypertension	[96]
NAC 500 mg/kg/day in drinking water	Maternal nicotine exposure	Gestational day 4 to postnatal day 10	SD/M	32	Prevented hypertension	[32]
Others						
Conjugated linoleic acid	Maternal high-fat diet	Pregnancy and lactation	SD/M	18	Attenuated hypertension	[97]
Fish oil	Maternal low protein diet	Pregnancy and 10 days after birth	Wistar/M &F	25	Prevented hypertension	[98]

Studies tabulated according to types of natural antioxidant, animal models and age at measure. STZ = streptozotocin. L-NAME = N^G^-nitro-L-arginine-methyl ester. CKD = chronic kidney disease. BCAA = branched-chain amino acid. LPS = lipopolysaccharide. TCDD = 2,3,7,8-tetrachlorodibenzo-p-dioxin. SD = Sprague-Dawley rat. SHR = spontaneously hypertensive rat. FHH = Fawn hooded hypertensive rat. M = male. F = female.
